# Vacuolar proteases and autophagy in phytopathogenic fungi: A review

**DOI:** 10.3389/ffunb.2022.948477

**Published:** 2022-10-26

**Authors:** Margarita Juárez-Montiel, Daniel Clark-Flores, Pedro Tesillo-Moreno, Esaú de la Vega-Camarillo, Dulce Andrade-Pavón, Juan Alfredo Hernández-García, César Hernández-Rodríguez, Lourdes Villa-Tanaca

**Affiliations:** Laboratorio de Biología Molecular de Bacterias y Levaduras, Departamento de Microbiología, Instituto Politécnico Nacional, Escuela Nacional de Ciencias Biológicas, Mexico City, Mexico

**Keywords:** phytopathogenic fungus, autophagy, *ATG8* and TOR, vacuolar proteases PrA and PrB, autophagic body degradation

## Abstract

Autophagy (macroautophagy) is a survival and virulence mechanism of different eukaryotic pathogens. Autophagosomes sequester cytosolic material and organelles, then fuse with or enter into the vacuole or lysosome (the lytic compartment of most fungal/plant cells and many animal cells, respectively). Subsequent degradation of cargoes delivered to the vacuole *via* autophagy and endocytosis maintains cellular homeostasis and survival in conditions of stress, cellular differentiation, and development. PrA and PrB are vacuolar aspartyl and serine endoproteases, respectively, that participate in the autophagy of fungi and contribute to the pathogenicity of phytopathogens. Whereas the levels of vacuolar proteases are regulated by the expression of the genes encoding them (e.g., *PEP4* for PrA and *PRB1* for PrB), their activity is governed by endogenous inhibitors. The aim of the current contribution is to review the main characteristics, regulation, and role of vacuolar soluble endoproteases and Atg proteins in the process of autophagy and the pathogenesis of three fungal phytopathogens: *Ustilago maydis*, *Magnaporthe oryzae*, and *Alternaria alternata*. Aspartyl and serine proteases are known to participate in autophagy in these fungi by degrading autophagic bodies. However, the gene responsible for encoding the vacuolar serine protease of *U. maydis* has yet to be identified. Based on *in silico* analysis, this *U. maydis* gene is proposed to be orthologous to the *Saccharomyces cerevisiae* genes *PRB1* and *PBI2*, known to encode the principal protease involved in the degradation of autophagic bodies and its inhibitor, respectively. In fungi that interact with plants, whether phytopathogenic or mycorrhizal, autophagy is a conserved cellular degradation process regulated through the TOR, PKA, and SNF1 pathways by ATG proteins and vacuolar proteases. Autophagy plays a preponderant role in the recycling of cell components as well as in the fungus-plant interaction.

## Introduction

Since fungal pathogens sense their nutrient supply and the changes in their environment, they respond with survival mechanisms aimed at establishing and promoting growth on their hosts. The course of invasion involves the remodeling of the fungal cell wall, adhesion, filamentation, appressorium formation, and sporulation. When first arriving to the surface of a plant, fungal pathogens face a nutrient-scarce environment and must constantly seek the appropriate mechanisms for survival. Particularly important are the mechanisms related to the release, transport, and metabolism of nutrients found during the different stages of infection (Johns et al., 2021).

**Figure 1:**
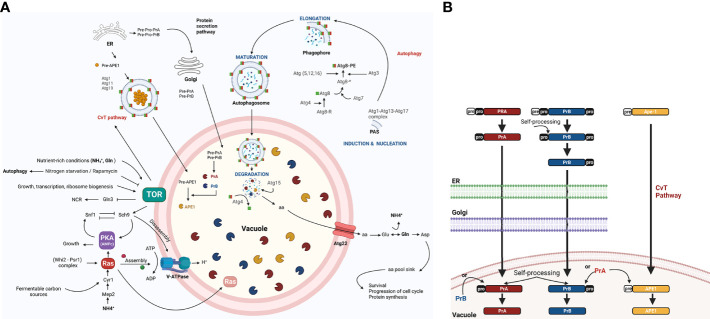
The role of autophagy, including the regulation of vacuolar proteases, in the yeast *S. cerevisiae*. **(A)** The TOR and RAS/cAMP kinases negatively regulate autophagy when the yeast is growing in nutrient rich conditions (glucose, NH^+^_4_, and Gln), thus avoiding G0 arrest. Both kinases inactivate Atg1, Atg13, and Atg8 proteins, which are involved in the first step of development of autophagosomes (Cebollero and Reggiori, 2009) and in the inactivation of transcription factor Gln3. The latter activates *NCR* genes and the Snf1 kinase. Under conditions of nutrient scarcity, Snf1 kinase is located in the vicinity of the vacuole and is released from the inhibition exerted by TOR and PKA, allowing it to inactivate TOR and PKA kinases. This inactivation takes place in part due to the targeting of Ras2 to the vacuole for proteolysis, mediated by the complex cell cycle regulator Whi2-phosphatase Psr1 (Leadsham et al., 2009). In addition to degrading specific proteins (e.g., Ras2), proteases and lipases break down autophagic bodies and their cargos, releasing amino acids and other biosynthetic units. These are either reused directly or induce Glu and Asp synthesis to replenish the amino acid sinks and therefore assure cell survival (Liu et al., 2021). In response to carbon availability, PKA and TOR regulate assembly and disassembly of V-ATPase through their effector Sch9 (Wilms et al., 2017). Vacuolar pH is regulated to maintain intracellular pH homeostasis and allow for the auto maturation carried out by protease PrA (Kane, 2006). **(B)** PrA matures to some extent in the endoplasmic reticulum (ER) before arriving to the vacuole and completing the process through automaturation. Subsequently, it stimulates the maturation of other vacuolar proteases, which are also synthesized as zymogens. Figure created by BioRender.com (accessed in April 2022).

Macroautophagy, hereafter called autophagy, is a highly conserved non-selective catabolic mechanism in eukaryotic cells consisting of the sequestering of cytosolic material (e.g., proteins and organelles) by autophagosomes and their delivery as autophagic bodies to the interior of the vacuole (for most fungal and plant cells) or their fusion with the lysosome (for many animal cells). A key element of autophagy is the delivery of hydrolytic enzymes into the vacuole (Fernandez et al., 2014; Zhu et al., 2019). After the membranes of autophagic bodies are degraded and the contents broken down by the vacuolar hydrolytic enzymes, degradation products are effluxed to the cytoplasm and reutilized in anabolic pathways (Parzych and Klionsky, 2019) (**Figure 1A**).

By degrading damaged molecules and organelles, autophagy facilitates the cellular homeostasis and at the same time their health and longer life span (Aman et al., 2021; Tyler and Johnson, 2018). During vegetative budding, DNA damage can induce cell cycle arrest and autophagy. Furthermore, the selective autophagy-related cytoplasm-to-vacuole targeting pathway (CvT) is also crucial for cellular homeostasis and operates during nutrient rich conditions (Levine and Klionsky, 2004). When facing a sufficiency of nutrients nutrient uptake, carried out by plasma membrane transporters of the cell, that participate in nutrient acquisition to sustain cellular growth and much of them also as receptor, are degraded *via* endocytosis to avoid an excess of nutrient uptake (Busto and Wedlich-Söldner, 2019).

Autophagy is induced under certain stress conditions, including nutrient deprivation, oxidative damage to organelles, cell cycle dysregulation, and critical levels of aging-derived molecules. It is suggested that the size of the insult determines whether autophagy favors cell survival or cell death. The latter option avoids the propagation of damaged or mutated cells (Azzopardi et al., 2017). Hence, the proper balance of autophagy seems to be vital. Whereas a dysfunction in autophagy leads to an accumulation of toxic subcellular components, resulting in aging and disease (Azzopardi et al., 2017; Aman et al., 2021), an excess of autophagy generates detrimental effects, as exemplified by its capacity to promote tumor cell survival and proliferation (Azzopardi et al., 2017).

Unlike in other organisms, autophagy is induced in yeasts, mainly by the signal of nutrient scarcity. The process is initiated by inhibiting its master negative regulator, the TOR kinase (target of rapamycin) (Noda and Ohsumi, 1998). Indeed, it is implicated in yeast survival during nutritional stress (Liu et al., 2021). The autophagic process is initiated by the inhibition of its master negative regulator, the TOR kinase (target of rapamycin) (Noda and Ohsumi, 1998). The RAS/cAMP pathway negatively regulates autophagy as well, while Snf11 stimulates this macromolecular turnover system (Cebollero and Reggiori, 2009; Shashkova et al., 2015).

Protein degradation and the subsequent efflux of amino acids by the transporter Atg22 allow the cell to directly reuse free amino acids to synthesize glutamate and aspartate through a series of deamination and transamination reactions (Liu et al., 2021). Poor nutrient conditions not only cause bulk degradation of proteins in the vacuole, but also degradation of specific proteins (**Figure 1A**). As a consequence of the specific degradation of active Ras2, for instance, the absence of this protein in the mitochondria prevents the activation of autophagic apoptosis (Leadsham et al., 2009).

The vacuolar proteolytic system of the yeast *Saccharomyces cerevisiae* encompasses at least nine different unspecific proteases. Endoproteases PrA and PrB (an aspartic and serine protease, respectively) (Takeshige et al., 1992) participate in the activation of various vacuolar hydrolases and thus play a well-documented role in autophagy. PrA and PrB are first synthesized as preproenzymes and then mature over a series of post translational modifications, such as proteolysis, that take place in the endoplasmic reticulum and the Golgi apparatus. After the arrival of PrA to a vacuole, the last step of processing occurs *via* pH-dependent automaturation. Subsequently, PrA matures PrB and other canonical vacuolar proteases. One example is aminopeptidase Ape1, which is targeted to the vacuole through the TOR-induced CvT pathway (Parzych and Klionsky, 2019) (**Figure 1B**). Although vacuolar proteases PrA and PrB were first described in *S. cerevisiae*, they have since been found in phytopathogens and other fungal models and, they take part in autophagic bodies and protein degradation, that increase from 40% to 85% under conditions of starvation compared to conditions of growth. This capacity is sharply reduced when impaired V-ATPase function alters vacuolar acidification (Kane, 2006).

**Figure 2:**
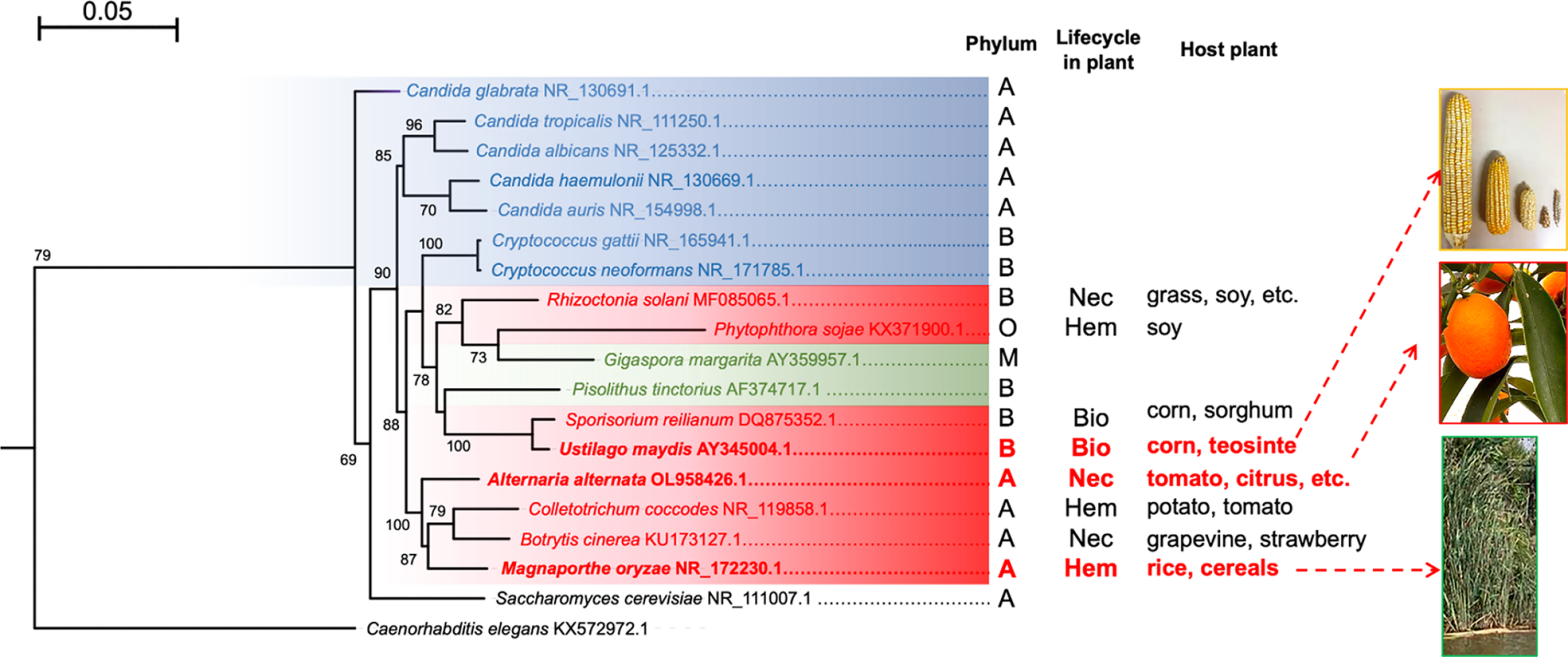
Maximum likelihood phylogenetic tree constructed with the sequences of the ITS regions of different fungi. Human pathogenic fungi are portrayed by blue branches, phytopathogenic fungi by red branches, and mycorrhizal fungi by green branches. *S. cerevisiae* and *C. elegans* are illustrated with black branches because the former is rarely isolated as a pathogen (its interest being purely biotechnological) and the latter served as an outgroup. The tree was generated with the MEGA11 program based on sequence alignment by using the MUSCLE algorithm, the substitution model HKY+G calculated by JModelTest, and 1,000 bootstrap replicates, as explained in the supplementary material. It was edited with the FigTree program. The phyla: A = Ascomycota, B = Basidiomycota, M = Mucoromycota, and O = Oomycota. The types of phytopathogen: Nec =necrotrophic, Bio = biotrophic, and Hem = hemibiotrophic. The host plant for each species is shown. The tree was generated with the MEGA11 program based on sequence alignment by using the MUSCLE algorithm, the substitution model HKY+G calculated by JModelTest, and 1,000 bootstrap replicates, as indicated in the supplementary material. It was edited with the FigTree program.

Autophagy has been extensively studied in yeasts such as *S. cerevisiae* and other ascomycetes, both pathogenic and non-pathogenic. However, there is less information *available* on filamentous fungi, and much less so regarding basidiomycete plant-interacting fungi. The current review aims to summarize the existing knowledge on autophagy, including its molecular basis of regulation *via* the TOR, PKA, and Snf1 kinases and by means of vacuolar proteases and transporters during the vacuolar efflux process. Furthermore, the biological function of vacuolar proteases in autophagy-dependent process such as morphogenesis and pathogenesis are explored. Regulation of proteolytic activity at different levels is also discussed, underlining endogenous protease inhibitors. These questions are discussed in relation to three phytopathogenic fungi: the hemibiotrophic *M. oryzae* and necrotrophic *A. alternata* of the Ascomycota phylum as well as the more distant biotrophic basidiomycete *U. maydis* (**Figure 2**). The conservation of autophagy is suggested to be beneficial for mycorrhizal fungi-plant symbiosis (Nehls and Plassard, 2018; Zhou et al., 2021), however they scape to the scope of this review.

### Autophagy in *Ustilago maydis*

Research on *Ustilago maydis* (the corn smut fungus) has provided insights into the *ATG* genes, the vacuolar proteases UmPrA and UmPrB, and the participation of such proteins in autophagy (Nadal and Gold, 2010; Soberanes-Gutiérrez et al., 2019), a well-conserved process in eukaryotes. To gain insights into the process of autophagy in *U. maydis*, it is necessary to first understand the context of the life cycle and pathogenesis of this fungus. Unlike other phytopathogenic fungi that are capable of infecting a wide variety of plants, *U. maydis* is a highly specific dimorphic phytopathogenic fungus only able to infect corn (*Zea mays*) and teosinte (*Zea mays* subsp. parviglumis). These two plants are closely related phylogenetically, likely sharing a common ancestor (Bruggeman et al., 2020).

**Figure 3:**
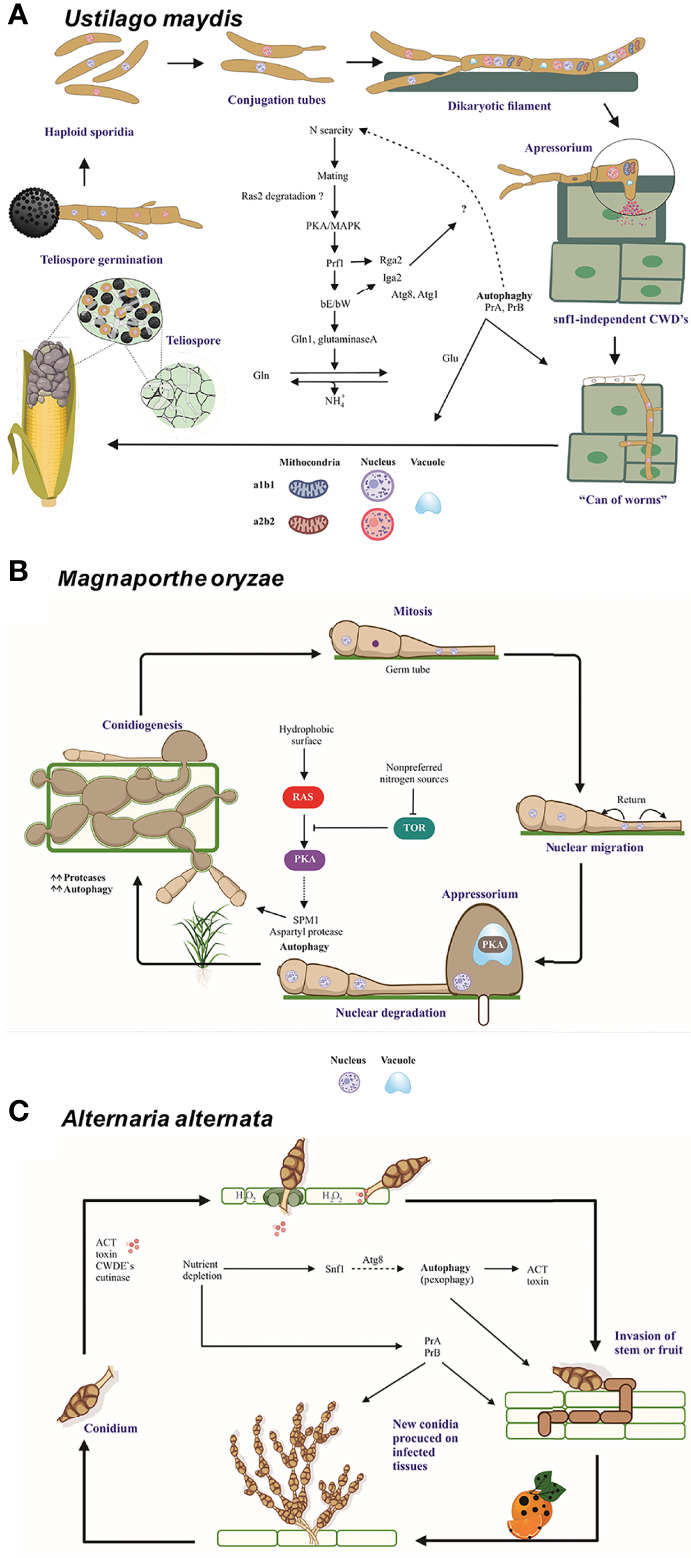
The role of autophagy and vacuolar proteases PrA and PrB in the pathosystem of *U. maydis*, *M. oryzae*, and *A alternata.* **(A)** In the course of an infection by *U. maydis*, nitrogen scarcity promotes filamentation of haploid sporidia mediated by the *bE* and *bW* genes (in an independent and nonredundant manner) and the Ump2 transporter, probably preparing the cells for mating (Wallen et al., 2021). After the mating of two compatible basidiospores, Rbf1 and bE/bW induce cell cycle arrest and dikaryotic filaments extend apically over the host tissue. Vacuolated areas separated from empty sections by septa are generated during filament extension. Subsequently, non-melanized appressoria develop and penetrate the plant cells by means of CWDE’s (Nadal et al., 2010). During this early stage, autophagy-related genes and vacuolar proteases *PRB1* and *PEP4* are overexpressed. Then the cell cycle restarts, fungal effectors are produced, and plant tissue is colonized. At the time of tumor formation, carbon, nitrogen and oligopeptide transporters, as well as autophagy-related proteins and proteases, all of them are overexpressed. Furthermore, the transcription factors *nit2* and *snf1* are upregulated (Lanver et al., 2018). In this late stage, there is a metabolic change in plant tissue that favors the growth of reproductive over vegetative tissue. Thus, carbon transporters Hxt1 and Suc1 are indispensable for fungal virulence (Schmitz et al., 2018). During the biotrophic phase, *U. maydis* faces conditions of nutrient stress and deploys many strategies to establish an effective infection system. **(B)** When three-celled conidia of *M. oryzae* arrive to the plant surface, the germ tube emerges, mitosis takes place, and the nuclei travel. As the appressoria mature, the conidia undergo autophagic programmed cell death to sustain appressorium function. The formation of appressoria is positively and negatively regulated by the Ras/cAMP and TOR pathways, respectively (Marroquin-Guzman et al., 2017), which in turn are regulated by the Whis2-Psr1 complex. The latter maintains the appropriate levels of cAMP and perhaps targets the Ras22 protein to the vacuole (Shi et al., 2021). **(C)** When the multicellular conidia of *A alternata* arrive to the plant tissue, they germinate and enter the plant cells through stomates or breaches by using an appressorium-like structure and CWDE’s. The plant cell membrane is immediately disrupted by ACT, which causes plant cell necrosis. Then the fungus proliferates and conidia are formed and released. The generation of H_2_O_2_ by the plant cell as a defense mechanism gives rise to pexophagy. The good functioning of peroxisomes is vital for the production of ACT toxin (Wu et al., 2021). Additionally, different aspects of autophagy are important for the pathogenicity of *A alternata*. The Δ*atg8* strain is unable of either to form aerial hyphae and provoke necrotic lesions, similarly to the Δ*prb* and a *pep4*-silenced strain. Interestingly PrB participating in the synthesis of secreted proteases (Fu et al., 2020). Nutrient scarcity leads to the expression of the entire autophagic machinery as well as the induction of Snf1, an essential protein for carbon utilization, vegetative growth, conidiation, and cell wall functions (Tang et al., 2020). Schemes were drawn whit BioRender.com (accessed in April 2022) and Corel Drawn v. 19.

*U. maydis* is a dimorphic fungus with a saprophytic and biotrophic phase during its life cycle. Its cycle begins with the germination of diploid teliospores followed by meiosis. Afterwards, haploid basidiospores are formed and present apical budding, allowing the sexually complementary yeasts to merge and establish a dikaryon, a stage in which the fungus can penetrate the maize plant tissue. The dikaryotic mycelium proliferates in the biotrophic phase of the fungus, and the fragmentation of the mycelium gives rise to dikaryotic teliospores that subsequently undergo karyogamy to generate mature melanized diploid teliospores. Damage to corn takes the form of galls or tumors (with a charcoal appearance) on the cob and other symptoms on the stem and leaves (chlorosis, increased anthocyanins, etc.) (Matei and Doehlemann, 2016) (**Figure 3A**).

### Orthologous genes of *ATG1* and *ATG8* in *U. maydis*

Carbon stress conditions in *U. maydis* favor the accumulation of autophagic bodies in the cell vacuoles as well as the overexpression of the genes *atg1* and *atg8* (Nadal and Gold, 2010). The autophagy genes *ATG1* and *ATG8* play an essential role in the induction of autophagy and the assembly of autophagosomes, respectively, in the nonpathogenic *S. cerevisiae* (**Figure 1A**). Strains of *U. maydis* lacking such genes have been evaluated (Nadal and Gold, 2010). According to the findings, the *atg8* gene of *U. maydis* is related to the process of autophagy in a manner similar to its orthologous gene *ATG8* of *S. cerevisiae*.

The deletion of *atg8* was observed in both the saprobic and biotrophic phases. The ∆*atg8* mutants lacked the accumulation of autophagosomes in the vacuole under conditions of carbon starvation, leading to reduced survival. There was an alteration of the characteristic apical budding and cell separation caused by the deletion of *atg8*, as shown by the structure of “lateral buds” of the yeasts in the exponential and stationary growth phase. The infection of maize plants with sexually complementary *atg8* mutant strains, versus wild-type (WT) strains, led to limited colonization of plants by the fungus, a decline in the number of plants that presented galls, and a diminished virulence index. Additionally, a relatively low density of invading hyphae was found in plant tissues as well as a decrease in the formation of teliospores.

**Figure 4:**
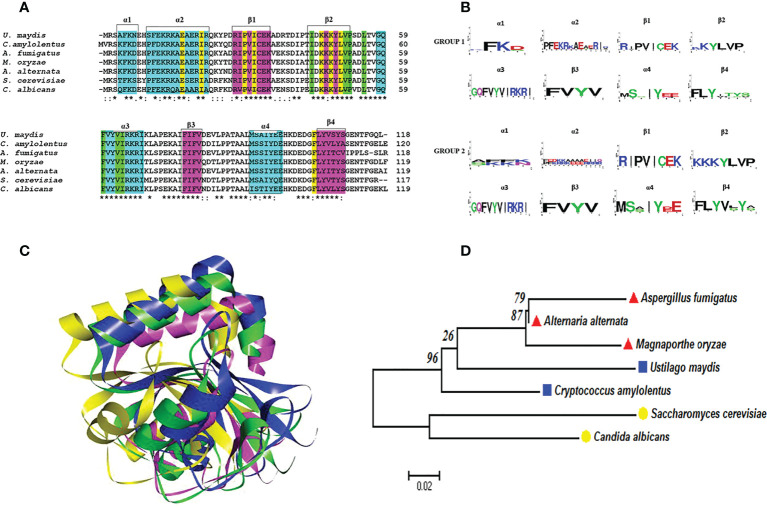
Atg8 proteins are conserved in pathogenic and nonpathogenic fungi. **(A)** Multiple alignments were performed with Clustal Omega. In the alignment, the amino acids associated with alpha-helical (cyan) or beta-strand (fuchsia) secondary structures are highlighted with boxes. Likewise, the important amino acid residues for the two deep hydrophobic pockets HP1 and HP2 function are highlighted in yellow and green, respectively. An asterisk (*) denotes a completely conserved residue; a colon (:) represents the conservation of the properties of the residual side chain; a dot (.) indicates residues with side chains of weakly similar properties; and a hyphen (-) designates a gap. **(B)** Consensus logos were generated with WebLogo. Group 1 (*S. cerevisiae*, *C albicans*, *A fumigatus*, *M. oryzae*, and *A alternata*) and group 2 (*C. amylolentus* and *U. maydis*). **(C)** 3D model of the Atg8 proteins. The overlap of *S. cerevisiae* (green) with the fungal phytopathogens is represented by utilizing the best model of the Atg8 of each fungus: *U. maydis* (blue), *M. oryzae* (fuchsia), and *A alternata* (yellow). The model was generated by homology modeler by using the Modeller 9.23 program, as explained in the supplementary material. **(D)** Phylogenetic analysis of the Atg8 of different organisms, carried out in the MEGA6 program with the maximum likelihood method, the WAG+G model, and 100 bootstrap replicates. The phylogenetic tree is drawn to scale, with the length of the branches depicting the corresponding evolutionary distances. Fungi that are grouped together in the same clade are portrayed with red, blue, and yellow symbols.

The Atg8 protein of *S. cerevisiae* undergoes post-translational processing at the C-terminus by the cysteine protease Atg4 to generate Atg8G116. The latter binds covalently to phosphatidylethanolamine (PE) in a ubiquitin-like reaction catalyzed by Atg3 and Atg7 (Ichimura et al., 2000; Kirisako et al., 2000). The analysis of the amino acid sequence of the protein deduced from the *U. maydis atg8* gene and its phylogenetic relationship with the sequences of other organisms (**Figure 4**) supports its characterization as the *ATG8* ortholog of *S. cerevisiae*, since the protein sequence of this basidiomycete contains the G116 residue in a conserved amino acid context (Nadal and Gold, 2010).

On the other hand, the deletion of the orthologous *atg1* gene of *U. maydis* resulted in phenotypes similar to the ∆*atg8* mutants during the saprobic phase. The *∆atg1* mutants were slightly less pathogenic for maize plants than the WT strains, although teliospore production was not affected. However, the phenotype of a double mutation at ∆*atg8* and ∆*atg1* was additive, evidenced by the even greater decline in pathogenicity as well as reduced teliospore production compared to the single-mutant strains. The mating process and the growth of filaments were not affected by the deletion of the *atg8* and *atg1* genes.

The importance of nutrient stress in triggering autophagy along with cellular adaptation and reprogramming in various organisms has been recognized for about two decades (Levine and Klionsky, 2004). For *U. maydis* and other fungi, carbon stress conditions existing at distinct stages of the fungal life cycle are able to cause cellular reprogramming, which can be provoked by a number of conditions. For example, the mating process involving the conjugation and fusion of two complementary sporidia of *U. maydis* is preferably carried out under conditions of starvation or a low nitrogen concentration (Wallen et al., 2021). Apart from its participation in the fungal response to stress conditions, autophagy might play a relevant role in the uniparental inheritance of a2 mitochondria in *U. maydis*. This is mediated by the small mitochondrial proteins Rga2 and Lga2, the encoding genes of which are located exclusively in the *a2* locus (Mendoza et al., 2020).

### Vacuolar protease A and autophagy in *U. maydis*

The *pep4* gene of *U. maydis* encodes a vacuolar aspartyl proteinase denominated UmPrA (also known as proteinase A or UmPep4). Its proteolytic activity, detected in the soluble fraction of cell extracts from *U. maydis*, is greater when the fungus is in the mycelial versus yeast phase. The presence of pepstatin, a specific inhibitor of aspartyl proteases, impedes the dimorphic transition from yeast to mycelium (Mercado-Flores et al., 2003).

According to proteomics studies, the protein encoded by the *pep4* gene in *U. maydis* is overexpressed during the yeast-mycelium transition, which is induced by the master regulator bE/bW. In addition to UmPep4, proteins orthologous to *S. cerevisiae* Gln1 and glutaminase A are overexpressed (Böhmer et al., 2007). These three proteins, involved in the later steps of autophagy in *S. cerevisiae*, carry out amino acid and glutamine synthesis under conditions of nutrient depletion (Liu et al., 2021). A similar strategy may exist for *U. maydis* to replenish N pools (**Figures 1A** and **3A**).

Mutants of *U. maydis* were generated in the *pep4* gene, creating a deficiency in protease A that diminished the dimorphic transition triggered by acid pH or by the use of fatty acids as the sole carbon source. As a consequence, the pathogenicity of the fungus towards the corn plant was affected. The *∆pep4* mutants caused a lower number of plants to be infected, a decrease in the signs of infection (e.g., chlorosis and an increase in anthocyanins), and less galls on the ears of corn (Soberanes-Guitiérrez et al., 2015). The construction of chimeric PrA proteins with green and red fluorescent proteins (PrA-GPF and PrA-RPF) confirmed the vacuolar location of the protease (Soberanes-Guitiérrez et al., 2015).

According to ultrastructural analysis of fungal cells, mutants of *U. maydis* deficient in PrA accumulate autophagosomes in the vacuole when incubated under conditions of carbon starvation stress (Soberanes-Gutiérrez et al., 2019). This is probably due to the inability of the yeasts to degrade autophagosomes. In the *S. cerevisiae* model, a similar phenomenon of autophagosome accumulation in vacuoles is observed in the absence of protease B or protease A (Takeshige et al., 1992).

Carbon or nitrogen starvation produces stress during the growth of *S. cerevisiae*, making a recycling system necessary. Autophagy is the primary transport pathway for bulk protein and organelle degradation (Teter and Klionsky, 2000), which occurs in the vacuole. The structure of the vacuole varies according to environmental conditions (Pratt et al., 2007). Autophagosomes collect material and enter the vacuole, where the enzymes of this organelle degrade their membrane, allowing for the recycling of the cytoplasmic-derived content (Abeliovich and Klionsky, 2001). In the mutants of *U*. *maydis* deficient in the acid proteinase UmPep4p (the OR1 and OR2 strains), the accumulation of autophagosomes was evident after nutrient shortage. CMAC staining revealed that the size and number of fusion events of vacuoles increased in complete medium for both WT and mutant strains as time progressed (Soberanes-Gutiérrez et al., 2019).

The *U. maydis pep4* gene has been cloned and heterologously expressed in order to biochemically characterize the recombinant protein. Based on its inhibition by pepstatin, the protein was found to be an aspartyl protease. Additionally, the tertiary structure of UmPrA turned out to be highly homologous to human cathepsin D, indicating that *Umpep4* could be the ortholog of the gene encoding human cathepsin D (Juárez-Montiel et al., 2018).

Interestingly, human cathepsin D, encoded by the *CTSD* gene, is active in the lysosome (the compartment equivalent to the fungal vacuole), and mutations in the gene result in protein accumulation in the organelle as well as nerve cell death (CLN10 disease). Genetic variants of the *CTSD* gene have been linked to neurodegenerative diseases such as Parkinson’s and Alzheimer’s disease (Bunk et al., 2021). Apart from degrading proteins, human cathepsin D can activate other proteins and regulate apoptosis (Di et al., 2021). The reduction in cathepsin D activity in human fibroblasts affects autophagic degradation, a change associated with insufficient lysosomal function (Tatti et al., 2012).

When detected in mammary secretions, human cathepsin D has been proposed as a cancer marker because its overexpression is related to tumor invasion. The overexpression of *PEP4* in *S. cerevisiae* has led to the presence of the enzyme in the nucleus of the yeast. This aspartyl protease may contribute to the degradation of nucleoporins during H_2_O_2_ stress (Kerstens and Van Dijck, 2018).

Taking all the prior evidence into account, the Pep4 protein from *U. maydis* is probably orthologous to human cathepsin D and Pep4 from *S. cerevisiae* (Juárez-Montiel et al., 2018). Furthermore, these vacuolar/lysosomal aspartyl proteases not only show a conserved tertiary structure with those of *Magnaporthe* and *Alternaria* but also a similar function (**Supplementary Figure 1**), reinforcing the idea of their evolutive relationship.

### Vacuolar protease B and autophagy in *U. maydis*

The activity of *U. maydis* protease B (UmPrBp), a serine protease, is inhibited by phenyl-methylsulfonyl fluoride (PMSF). The latter enzyme was detected in *U. maydis* for the first time in 2003, using Hide–Remazol Brilliant Blue R (Hide powder azure) substrate at pH 7 and in the presence of an endogenous inhibitor activated and released at acidic pH (Mercado-Flores et al., 2003). Considering that the inhibition of UmPrB with PMSF led to the accumulation of autophagosomes, this serine protease (like UmPrA) likely participates in the degradation of autophagic bodies (Soberanes-Gutiérrez et al., 2019).

The absence of protease B during the process of autophagy in *S. cerevisiae* causes an accumulation of autophagosomes in the vacuoles (Takeshige et al., 1992). After autophagosome formation and the transportation of the cellular cargoes to vacuoles, autophagic membranes are degraded by vacuolar enzymes, allowing the cellular components inside to meet the nutritional requirements of *S. cerevisiae* under conditions of starvation (Takeshige et al., 1992; Abeliovich and Klionsky, 2001).

In *S. cerevisiae* cells under conditions of carbon or nitrogen starvation, and in mutants deficient in protease A and protease B, ribosomes and mitochondria are observed inside of autophagosomes. Hence, these vacuolar proteases and the process of autophagy in general seem to degrade and recycle intracellular components (Takeshige et al., 1992). In WT *U. maydis*, autophagic bodies did not accumulate in the cell vacuoles until the serine protease inhibitor PMSF was added. In contrast, such accumulation occurred in sporidia with a mutation in the acid proteinase Pep4p even in the absence of PMSF. Overall, the findings provide evidence of a key role for PrB in the degradation of autophagic bodies in *U. maydis*. In addition, PMSF-treated WT FB2 cells were shown to accumulate autophagic bodies, suggesting that vacuolar proteinase B is involved in the degradation of the autophagic bodies in other systems as well. In the same sense, autophagic bodies have been reported to disappear if cells are transferred to a culture medium without PMSF (Takeshige et al., 1992; Baba et al., 1994).

**Figure 5:**
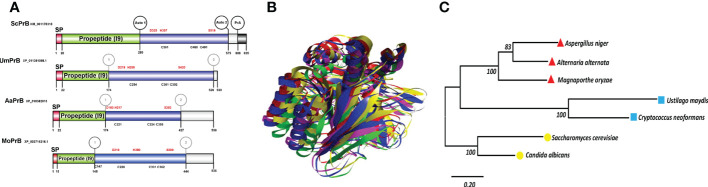
Serine vacuolar endoproteases PrBs are conserved in pathogenic and nonpathogenic fungi. **(A)** The characteristic domains of vacuolar proteases of the S8 family are illustrated, including the DHS catalytic triad of serine proteases, the probable maturation sites, and the I9 inhibitor domain of the propeptide (predicted in the Expasy server). Proteases such as ScPrB are apparently synthesized as zymogens, which are inactive until propeptides are removed in the vacuole. The cysteines in the protein are also shown. The circles in the *S. cerevisiae* protease designate the regions where autocatalysis and PrA processing are conducted. In the rest of the proteases, the circles denote regions of theoretical maturation. The alignment of sequences was performed with Clustal X and the drawing with BioRender.com (accessed in April 2022). **(B)** Tertiary structure of fungal PrBs. The overlap is represented by utilizing the best model of PrB of *S. cerevisiae* (green) and of the three fungal phytopathogens herein studied: *U. maydis* (blue), *M. oryzae* (fuchsia), and *A alternata* (yellow). **(C)** Phylogenetic analysis of the PrB of different organisms. Carried out in the MEGA6 program with the maximum likelihood method, the WAG+G model, and 100 bootstrap replicates. The phylogenetic tree is drawn to scale, with the length of the branches depicting the corresponding evolutionary distances. Fungi that are grouped together in the same clade are portrayed with red, blue, and yellow symbols.

As already mentioned, the vacuolar proteases PrA and PrB of *S. cerevisiae* undergo distinct proteolytic processes from the beginning of their synthesis to their passage through the secretion route *via* the endoplasmic reticulum-Golgi apparatus-vacuole (Parzych and Klionsky, 2019). Whereas PrB from *S. cerevisiae* needs 4 post-translational processing sites to become a mature enzyme (**Figures 1B**, **5**), the proteolytic processing of the putative proteinase B of *U. maydis*, encoded by the gene with access number Um4400, is herein proposed to encompass only three such sites (**Figure 5**). Given its carboxyl terminal portion, UmPrB may not mature until it reaches the vacuole. At this time, automaturation likely takes place rather than a process directly involving UmPrA. Moreover, the predicted protein presents the characteristic domain of a subtilisin-like serine protease of the S8 family. Even though *Magnaporthe* and *Alternaria* are ascomycete fungi, processing sites similar to those of UmPrB are found in their respective PrB proteins, as are similar domains and tertiary structures. Furthermore, the phylogenetic relationship of UmPrB with the PrB of other related fungi indicates the probable orthology between them (**Figure 5**).

Both PrA and PrB propeptides inhibit their cognate proteases until the latter are removed upon reaching the vacuole. The endogenous cytoplasmic inhibitors for these proteases are known, AI3 and PBI2 (I2B, YIB2) for PrA and PrB, respectively (Dunaevsky et al., 2014). The inhibitory activity of PrB propeptide and the PBI2 inhibitor depends on their C-terminal region. It has been determined that the C-terminal region of the propeptide of *S. cerevisiae* PrB and the inhibitor PBI2 share conserved regions with the subtilisin BPN´ propeptide, which inhibits its cognate protein. All these inhibitors belong to the I9 family, which lacks disulfide bonds, an unusual characteristic of serine protease inhibitors (Kojima et al., 1999). Apart from its inhibitory function, PBI2 participates in vacuole inheritance (together with thioredoxin) by regulating vacuole coalescence. Thus, inhibitor-protease binding may be a regulating mechanism of the latter process (Slusarewicz et al., 1997).

**Figure 6:**
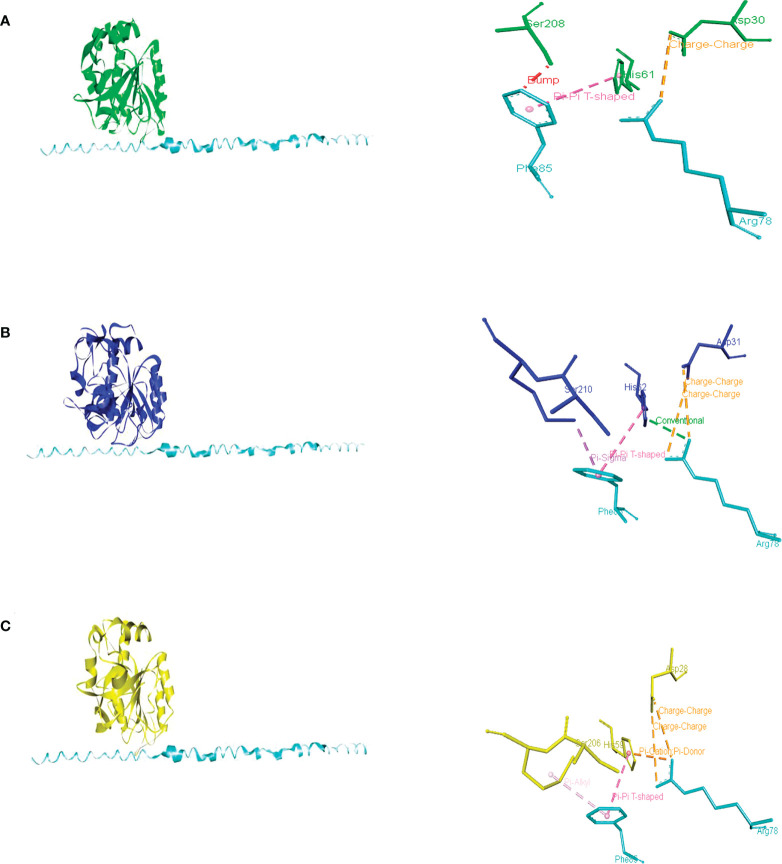
Intermolecular interactions of inhibitory peptide Um10059 (Um2129) (cyan) with PrBSc **(A)**, PrBUm **(B)**, and PrBAa **(C)**. Dotted lines indicate the type of interactions. For the purpose of clarity, the interactions between the amino acid residues of the PrB catalytic triad (Asp, His, and Ser) and the inhibitory Um10059 are shown. The interactions with *M. oryzae* were not shown since none of the residues of the catalytic triad interact with the inhibitor of *U. maydis*.

An AI3 homolog has not been identified in *U. maydis*, though a probable PBI2 ortholog was detected (with access number Um10059, JGI portal Um02129). Whereas the predicted protein contains a unique region not present in PrB propeptides or PBI2, its C-terminal region is conserved (**Supplementary Figure 2**). The docking of this peptide with PrB of *Saccharomyces*, *Ustilago*, and *Alternaria* evidenced its interaction with similar amino acid residues in each case, including the ones that make up the catalytic triad (Asp, His, and Ser) (**Figure 6**). The PBI2 of *S. cerevisiae* interacts with most of the same catalytic residues of these PrBs (**Supplementary Figure 3**). Similar interactions have been seen for the subtilisin of *B. amyloliquefaciens* (Gallagher et al., 1995). The protein Um10059 might be responsible for the inhibition of the UmPrB activity reported by Mercado-Flores et al. (2003).

According to the docking analysis, there are distinct characteristics of specificity of the propeptides, and such differences are related to the phylum of the fungi. While the PrB propeptide of *S. cerevisiae* was able to interact with the catalytic residues of PrB of *Saccharomyces*, *Magnaporthe*, and *Alternaria*, the PrB propeptide of *Ustilago* seems to be different because it could only interact with PrB residues distinct from the catalytic ones (**Supplementary Figure 4** and **Supplementary Table 2**).

In addition to the biological implication of these inhibitors, interesting biotechnological applications have been suggested in medicine (e.g., to treat cancer and other diseases) and agriculture (e.g., to target phytopathogens) (Dunaevsky et al., 2014. Applications of inhibitors are discussed later in this review.

### Atg8 and Atg1 proteins and autophagy in *Magnaporthe oryzae*

*Magnaporthe oryzae* (anamorph: *Pyricularia oryzae*) is a hemibiotrophic ascomycete and the causal agent of rice blast disease. It develops a highly melanized dome-shape appressorium from a three-celled conidium (spore) (Wilson and Talbot, 2009). After germination of *M. oryzae* conidia, mitosis and nuclear migration occur. Then the nuclei of the conidia are degraded *via* autophagy to sustain the successful formation of infectious hyphae (**Figure 3B**).

Under nutrient-poor conditions, Δ*atg8* mutants did not exhibit autophagic activity. The accumulation of autophagic bodies was not seen inside vacuoles in mycelium cells (using mono-dansyl cadaverine stain), even when PMSF (an inhibitor of serine proteases) was added. The structure of the *M. oryzae* Atg8 protein is similar to the one existing in *S. cerevisiae* Atg8 (**Figure 4**), and there was no nuclear breakdown in the Δ*atg8* mutant that was capable of affecting pathogenesis without affecting its capacity to produce appressoria (Veneault-Fourrey et al., 2006; Veneault-Fourrey and Talbot, 2007).

A mechanical force derived from the high osmotic pressure inside the appressorium facilitates the disruption of the plant cuticle (**Figure 3B**). Since this pressure is favored by glycerol accumulation and melanization, it causes cytoskeleton reorientation and polarization, leading to the formation of the penetration peg (Osés-Ruiz and Talbot, 2017). In *M. oryzae*, deletion of the *ATG14* genes altered autophagy, glycogen mobilization, the number of lipid droplets, and turgor pressure, thus affecting infection and conidiation (Liu et al., 2017). Bulk autophagy of the material of the conidium plays a major role in the turgor pressure, melanization, and repolarization of appressoria. However, glycogen mobilization is not essential in the generation of turgor pressure. Rather, the degradation of lipid droplets seems to be the main source of glycerol and melanin (Foster et al., 2017).

### Vacuolar protease A and autophagy in *M. oryzae*

During appressorium formation induced on a hydrophobic GelBond surface, two aspartyl proteases genes, *MGG_09351.5* and *MGG_00981.5*, were overexpressed. Similar results were obtained when the development of appressoria was triggered by the addition of cAMP. In either case, the most upregulated genes were generally related to protein and amino acid degradation as well as carbohydrate and lipid metabolism, while genes linked to protein synthesis tended to be downregulated (Oh et al., 2008). On the other hand, the most probable vacuolar aspartyl protease shows a conserved 3D structure in relation to the PrA from other yeasts and filamentous fungi and to human cathepsin D (**Supplementary Figure 1**). Hence, all of them may be homologous and implicated in the last step of autophagy. Overall, this information highlights the crucial function of aspartyl proteases in eukaryotic organisms.

### Vacuolar protease B and autophagy in *M. oryzae*

*SPM1* (MGG_03670.5) in *M. oryzae* encodes the endoprotease Spm1, related to PrB in *S. cerevisiae* (**Figure 5**), which is targeted to the vacuole in the course of an infection. Spm1 is upregulated at the time of appressorium formation. Although mutants produced melanized appressoria in the presence of cAMP and under conditions of nitrogen starvation in synthetic media, a decrease was found in virulence, aerial hyphae, and conidiation (Oh et al., 2008). Spm1 is involved in multiple aspects of the infection process such as conidia germination, invasion, and endocytosis. As evidence of its role in endocytosis, *spm1* mutants were not capable of sustaining the vacuolar accumulation of FM4-64. In agreement with the canonical function of the vacuolar serin endoproteases, deletion of the *SPM1* gene leads to the accumulation of granular particles inside the vacuoles of appressoria and conidia (Saitoh et al., 2009).

### Atg8 and Atg1 proteins and autophagy in *Alternaria alternata*

*Alternaria alternata* (an ascomycete) is able to cause disease in a plethora of hosts by diverse pathotypes, each of which secretes a host-specific toxin (HST) crucial for successful infection. For instance, the tangerine pathotype produces adenylate cyclase toxin (ACT), an HST for *Citrus reticulata*, *Citrus sinensis*, and *Citrus paradisi*. ACT is responsible for the necrotic lesions on the leaves and fruit, typical symptoms of citrus brown spot (Kohmoto et al., 1993). Unlike biotrophic and hemibiotrophic fungi, necrotrophic pathogens such as the tangerine pathotype of *A. alternata* kill their host before colonization. This is done by generating ACT and cell wall degrading enzymes (CWDE’s) and occurs immediately after penetrating the plant through stomata on the abaxial side of the leaves as well by a small non-melanized appressorium on the adaxial side (**Figure 3C**).

As a defensive response to fungal infection, a plant is to produce a great quantity of H_2_O_2_ (the hypersensitive response). However, necrotrophic pathogens have developed mechanisms to cope with H_2_O_2_ stress (Lin et al., 2011). For example, the tangerine pathotype of *A. alternata* activates Yap1 and Tfb5 transcription factors, regulator Skn7, HSK and Hog1 kinases, catalase, and superoxide dismutase (SOD). Fe^+^, the SOD cofactor, is taken from the environment through siderophores and stored in vacuoles. Whereas the secretion of siderophores is indispensable for the pathogenicity of *A. alternata*, it is dispensable for the pathogenicity of *U. maydis* (Chung, 2012). On the other hand, the regulation of Fe^+^ *via* vacuolar compartmentalization is unknown in the latter biotrophic fungus.

Peroxisomes are usually involved in the elimination of H_2_O_2_. Nevertheless, subsequent to H_2_O_2_ treatment of *Alternaria*, the level of peroxisomes declines and they are found colocalized with vacuoles, suggesting that pexophagy is taking place. The alteration of the function of peroxisomes in *Alternaria* Δ*pex6* mutant affects pathogenicity and autophagy, partially restored adding purified ACT toxin. In Δ*pex6* mutants, there was reduced expression of *ATG8*, a gene responsible for encoding the Atg8 protein, which accumulates in vacuoles and participates in autophagosome formation when *A. alternata* grows in the absence of nitrogen (Fu et al., 2020; Wu et al., 2021). Moreover, this Atg8 has a highly conserved tertiary structure in relation to the homologous proteins of the other fungi described herein (**Figure 4**). Overall, the evidence supports the notion that the function of Atg8 could be similar in fungi, as has been proposed in previous reports (Ivory et al., 2021).

In *A. alternata*, as in diverse yeasts and filamentous fungi, macroautophagy appears to be important for maintaining the homeostasis of cells under conditions of stress. The pathway might be similar, involving factors such as Atg proteins, TOR, Snf1, PKA, and MAPK kinases.

### Vacuolar protease A and autophagy in *Alternaria alternata*

The crucial role of the aspartyl protease in the course of plant infection by the *A. alternata* tangerine pathotype was demonstrated in a *pep4* gene silencing assay. The downregulation of *pep4* gave rise to the incapacity of *A. alternata* to produce conidia in PDA medium, and the resulting small necrotic plant lesions indicated reduced virulence. In the silenced *pep4* strain, autophagic bodies accumulated inside the vacuole, revealing the essential participation of the corresponding protein in autophagy (Fu et al., 2020). This information together with the similarity between the predicted structure of PrA of *A. alternata* with the structure of the same protein in other fungi (**Supplementary Figure 1**) suggests that all the vacuolar aspartyl proteases discussed herein are orthologous proteins.

### Vacuolar protease B and autophagy in *A. alternata*

In addition to the canonical vacuolar aspartyl protease in *A. alternata*, a subtilisin-like serine protease of the S8 family has also been detected. Based on the domains identified and the physical interaction with PrA, demonstrated by using a yeast two-hybrid assay (Fu et al., 2020), as well as the similar maturation and probable self-regulation (by the I9 domain of the propeptide of PrB of *S. cerevisiae*) these two proteins are probably orthologous. During incubation in minimal medium, growth was slower for *A. alternata* Δ*prb1* strains than the WT strain. Normal growth was restored by adding citrus leaves, yeast extract, and urea as a nitrogen source, but the mutant was not capable of normal conidiation even in PDA medium. Although *A. alternata* Δ*prb* exhibited decreased pathogenicity compared to the WT strain (evidenced by the small necrotic lesions it induced), its production of ACT was not affected. Similar to PrA in *A. alternata*, its PrB is essential for breaking down vacuolar autophagic bodies in conditions of nutrient scarcity (Fu et al., 2020). It is similar in structure to other PrBs and is phylogenetically related to them (**Figure 5**). Furthermore, it is inhibited by the predicted *U. maydis* Um00159 protein and the PBI2 inhibitor (**Figure 6C** and **Supplementary Figure 3D**).

Microbial virulence, a measure of the capacity of a given microbe to cause damage in a susceptible host, can be enhanced, lost, and restored. While the virulence of phytopathogenic fungi depends on secreted proteins such as effectors and toxins, cellular metabolism, morphogenesis, and sporulation are also involved (Johns et al., 2021). The relationship between the process of autophagy in phytopathogenic fungi and their virulence has been established. The phytopathogenic fungi *U. maydis*, *M. oryzae*, and *A. alternata* lacking the vacuolar proteases PrA and PrB and the subtilisin-like protease Spm1 were deficient in terms of autophagy, morphological transitions related to the infection processes, and probably the secretome (Saitoh et al., 2009; Soberanes-Gutiérrez et al., 2015) (**Figure 3**).

### Possible applications derived from the study of autophagy

As already mentioned, the process of autophagy has been investigated in humans and other mammals, and its role in the pathogenesis of neurodegenerative diseases and cancer has been demonstrated. Inhibitors and activators of autophagy could serve to better understand the regulation of this process in human diseases. The testing of autophagy-targeted drugs has also been proposed (Yang et al., 2013). Given that the process of autophagy is highly regulated, a balance must exist between its induction and inhibition (Meijer and Codogno, 2009). According to several authors, activators of autophagy may have therapeutic benefits for various diseases (Yang et al., 2013). Among such activators are endoplasmic reticulum stress inducers (Ciechomska et al., 2013), rapamycin and its derivatives (Ravikumar et al., 2004), trehalose (Sarkar et al., 2007), and inositol monophosphatase (IMPase) inhibitors (e.g., lithium chloride) (Sarkar et al., 2005). On the other hand, autophagy can be suppressed at any of its stages. One of the problems is the lack of specificity of many autophagy-inhibiting compounds. The following compounds have been posed as autophagy inhibitors: PI3K inhibitors (Castino et al., 2010); cycloheximide (Oliva et al., 1992), vacuolar-type H(+) ATPase inhibitors (Wu et al., 2009), lysosomal lumen alkalizers (Harhaji-Trajkovic et al., 2012), and acid protease inhibitors (e.g., leupeptin and pepstatin A) (Kominami et al., 1983; Tanida et al., 2005). In addition to contemplating pharmacological modulation of autophagy based on the type and stage of certain diseases, gene-targeting approaches have been postulated as a new therapeutic option for human diseases associated with the deregulation of the process of autophagy (Yang et al., 2013).

The inhibition of autophagy in human pathogenic fungi is an approach worthy of investigation since it represents a mechanism distinct from conventional antifungals and thus could plausibly help to combat the multi-resistance problem of yeasts of the genus *Candida*, which has become a public health problem in recent years (Madrigal-Aguilar et al., 2022). The participation of the ATG1 and ATG11 genes in virulence and autophagy has been assessed in *Candida glabrata* and *Candida albicans* (Cui et al., 2019; Shimamura et al., 2019), as has the role of vacuolar proteases in conditions of nutritional stress and their possible association with the process of autophagy (Sepúlveda-González et al., 2016; Cortez-Sánchez et al., 2018). Future research is needed into the application of inhibitors or activators of autophagy in phytopathogens and fungi pathogenic to humans in order to reduce or avoid the effects on the corresponding hosts.

## Conclusions

The current review focuses on the role of vacuolar proteases PrA and PrB, the Atg8 and other molecular players in the process of autophagy of three phytopathogenic fungi (belonging to two phyla) with different plant-interaction lifestyles. The insights provided may be useful for the regulation of the process of autophagy in both the host and pathogenic fungi. Included in the study were a biotrophic basidiomycete (*U. maydis*), a hemibiotrophic ascomycete (*M. oryzae*), and a necrotrophic ascomycete (*A. alternata*). The plausible orthology between the vacuolar proteases PrA and PrB in such fungi and in *S. cerevisiae* is suggested by both molecular phylogeny and their respective functions, as they all participate in the degradation of autophagic bodies in the vacuole during the process of autophagy. Moreover, the catalytic domains are conserved in the primary and tertiary structures of the proteins from the three species of phytopathogenic fungi herein examined. According to the molecular modeling analysis, the endogenous inhibitor of PrB, denominated PBI2, exists not only in *S. cerevisiae* but probably also in *U*. *maydis.* This inhibitor regulates the proteolytic activity of PrB. PBI2 and Um10059 each bind to the catalytic residues of their corresponding protease and those of the *Alternaria* protease. Likewise, the Atg8 protein is conserved in these phytopathogenic species. Based on the analyses of the amino acid sequences of the corresponding proteins of *U. maydis*, *M. oryzae*, and *A. alternata*, they are probably orthologs of *S. cerevisiae* Atg8. Overall, the process of autophagy seems to be conserved in phytopathogenic fungi, in non-pathogenic fungi such as mycorrhizae, and in other eukaryotic organisms (e.g., mammals). The fact that, unlike the propeptide of PrB, the putative PrB of *U. maydis* does not recognize the catalytic residues of its respective serine protease or those of the serine proteases of other fungi is likely due to adaptation and the evolutive distance between the organisms (ascomycetes and basidiomycetes). The function of vacuolar proteases PrA and PrB and of autophagy in general is part of the cell response to nutritional stress in order to replenish critical constituents needed for survival. Under basal conditions, autophagy removes long-lived, damaged, and redundant proteins or organelles to allow for cellular longevity. It serves as a type of programmed cell death in the course of cell development and differentiation. In the process of infection, fungal phytopathogens sense nutritional scarcity and other environmental conditions through the cAMP/PKA and MAPK signaling pathways. These pathways regulate the nutrient sensor TOR as well as the Atg proteins that initiate autophagy and participate in the distinct stages of fungal development of the three phytopathogenic fungi presently examined. During the stages of spore germination, mating, penetration of the host plant, and sporogenesis, it is crucial for the fungus to maintain its virulence and pathogenicity. Hence, autophagy plays a major role in cell biology from the first to the last steps of the process.

## Author contributions

LV-T and MJ-M conceived of the project and wrote the manuscript. DC-F, PTM, and EC performed the phylogenetic analysis of the fungi and the vacuolar proteases. DA-P, JH-G, and CH-R carried out docking and the bioinformatic analysis of proteins as well as the evolutive analysis. All authors contributed to the article and approved the submitted version.

## Funding

This work was supported by SIP-IPN grants (20220742, 20220795, and 20221723).

## Acknowledgments

DC-F, PT-M and EC appreciate the graduate scholarship awarded by CONACyT as well as the scholarship complements furnished by the SIP-IPN (BEIFI). The authors would like to thank BLfor proofreading the manuscript. MJ-M, CH-R, and LV-T are fellows of the Estímulos al Desempeño de los Investigadores (EDI-IPN) and Comisión de Operación y Fomento de Actividades Académicas (COFAA-IPN) programs.

## Conflict of interest

The authors declare that the research was conducted in the absence of any commercial or financial relationships that could be construed as a potential conflict of interest.

## Publisher’s note

All claims expressed in this article are solely those of the authors and do not necessarily represent those of their affiliated organizations, or those of the publisher, the editors and the reviewers. Any product that may be evaluated in this article, or claim that may be made by its manufacturer, is not guaranteed or endorsed by the publisher.
