# Supplementary material for: Vacuolar proteases and autophagy in phytopathogenic fungi: A review
Source: Front Fungal Biol. 2022 Oct 26;3:948477. doi: 10.3389/ffunb.2022.948477 (PMC10512327; doi:10.3389/ffunb.2022.948477)
Supplement: Supplementary file 1 [file DataSheet_1.pdf]

SUPPLEMENTARY MATERIAL

Vacuolar proteases and autophagy in phytopathogenic fungi: a review

Margarita Juárez-Montiel, Daniel Clark-Flores, Pedro Tesillo-Moreno, Esaú de la Vega Camarillo, Dulce Andrade-Pavón, Juan Alfredo Hernández-García, César Hernández-Rodríguez, Lourdes Villa-Tanaca

Laboratorio de Biología Molecular de Bacterias y Levaduras. Departamento de Microbiología, Escuela Nacional de Ciencias Biológicas, Instituto Politécnico Nacional, Mexico City, Mexico.

Materials and Methods

Phylogenetic tree of fungi showing evolutionary relationships, using the ITS region

The phylogenetic tree was generated by using the MEGA11 program and subsequently edited with FigTree (Tamura *et al*., 2021). Sequence alignment and editing was done in the SeaView program with the Muscle algorithm. The nucleotide substitution model employed was HKY calculated by JModelTest (Posada, 2008). The final tree was created with gamma distribution based on 1,000 bootstrap replicates and the maximum likelihood method (Morrison, 2007). The nucleotide sequences were downloaded from the NCBI database (https://www.ncbi.nlm.nih.gov/): *Candida auris* (NR_154998.1), *C. haemulonii* (NR_130669.1), *C. albicans* (NR_125332.1), *C. tropicalis* (NR_111250.1), *C. glabrata* (NR_130691.1), *Saccharomyces cerevisiae* (NR_111007.1), *Cryptococcus neoformans* (NR_171785.1), *C. gattii* (NR_165941.1), *U. maydis* (AY345004.1), *Sporisorium reilianum* (DQ875352.1), *Magnaporthe oryzae* (NR_172230.1), *Alternaria alternata* (OL958426.1), *Phytophthora sojae* (KX371900.1), *Gigaspora margarita* (AY359957.1), *Pisolithus tinctorius* (AF374717.1), *Caenorhabditis elegans* (KX572972.1), *Colletotrichum coccodes* (NR_119858.1), *Rhizoctonia solani* (MF085065.1), and *Botrytis cinerea* (KU173127.1).

Identification of conserved sequences and phylogenetic analysis of Atg8 and PrB proteins from different organisms

The amino acid sequences for the Atg8 and PrB of the following organisms were downloaded from the NCBI database: *S. cerevisiae* (NM_001178318) (NM_001178318), *C. albicans* (XP_019330653) (XP_715244), *Aspergillus fumigatus* (KAH3637871) and *A. niger* (XP_001391470.1), *Magnaporthe oryzae* (ACJ06588) (XP_003716216.1), *Alternaria*

alternata (XP_018382869) (XP_018382013), *Cryptococcus amylolentus* (XP_018996636) and *C. neoformas* (OXG28165.1) and *U. maydis* (XP_011391873) (XP_011391098). Once the sequence of each enzyme was downloaded, it was selected and subjected to multiple alignment in the Clustal Omega program (Aiyar, 2000). The motif domains were located and visualized with the web server WebLogo (http://weblogo.berkeley.edu/logo.cgi). Based on the sequences, a phylogenetic tree was constructed on the MEGA6 program (Tamura *et al*., 2013) by utilizing the maximum likelihood method and the WAG + G model. Finally, 100 bootstrap replicates were used to evaluate the reliability of the phylogenetic tree.

Generation of 3D PrA, PrB, and Atg8 from *S. cerevisiae* and fungal phytopathogens through homology modeling

The following sequences were downloaded from the NCBI database (http: //www.ncbi .nlm.nih.gov) (Sharma *et al*., 2018): the PrA enzyme of *Homo sapiens* (NP_001900); PrA, PrB, and Atg8 of *S. cerevisiae* (NP_010854, NM_001178318, and NM_001178318), *M. oryzae* (XP_003718037, XP_003716216.1, and ACJ06588), *A. alternata* (XP_018380546, XP_018382013, and XP_018382869) and *U. maydis* (XP_011391245, XP_011391098, and XP_011391873). The percentage of identity was determined for each of the sequences of PrB and Atg8 of fungal phytopathogens, as well as of the target PrB protein of *Bacillus amyloliquefaciens* and Atg8 of *S. cerevisiae* with the Emboss Water server. The sequences were used for generating the 3D models with the homology modeling technique on the Modeller 9.23 program (Webb and Sali, 2014), with the crystallized structures of *S. cerevisiae* (PDB: 1FMX), *B. amyloliquefaciens* (PDB: 1S01), and *S. cerevisiae* (PDB: 6WY6), deposited in the protein data bank (http://www.rcsb.org/pdb/) (Berman *et al*., 2000), serving as templates. The models were overlapped in the Discovery Studio Visualizer. Among the 3D models obtained for PrA, PrB, and Atg8, the one with the minimum score was chosen and evaluated with the discrete optimized protein energy method (DOPE) (Shen and Sali, 2006).

Protein-peptide interactions

The study of protein-peptide interactions was carried out with the best 3D models of PrB proteins from *S. cerevisiae*, *U. maydis*, *M. oryzae*, and *A. alternata* previously afforded by the homology modeling technique. Likewise, the amino acid sequences of the peptides (PBI2 and Um10059) and propeptides (PrB1 and Um4400) in FASTA format were transformed into PDB with the Open Babel GUI program (O'Boyle *et al*., 2011). Subsequently, the PrBs and peptide-propeptides mentioned were subjected to a protein-protein interaction study with the HDOCK server (Yan *et al*., 2020). The results of the interactions were described and organized in tables, and the graphs of such interactions were elaborated in the Discovery Studio Visualizer.

Results

Conservation of the Atg8, PrA, and PrB proteins from different organisms

After retrieving the protein sequences of the organisms mentioned in the previous section, the percentage of identity and similarity was determined **(Supplementary Table 1).** These values suggests that the Atg8, PrA, and PrB proteins of the phytopathogens herein studied are likely to be conserved**.**

Supplementary Table 1. Percentage of identity and similarity of the sequences of the PrA, PrB, and Atg8 proteins of *S. cerevisiae* with the fungal phytopathogens.

Protein	Organism	Model	% identity	% similarity
**PrA**	*S. cerevisiae*	*U. maydis*	63.6	75.5
		M. oryzae	63.4	79.9
		A. alternata	67.1	82
		H. sapiens	44.1	59.4
**PrB**	*S. cerevisiae*	*U. maydis*	63.5	74.5
		M. oryzae	68.7	81.5
		A. alternata	71.5	80.8
**Atg8**	*S. cerevisiae*	*U. maydis*	77.9	92
		M. oryzae	80.5	89.4
		A. alternata	79.6	90.3

3D models of PrA, PrB, and Atg8 from *S. cerevisiae* and the fungal phytopathogens (*U. maydis, M. oryzae,* and *A. alternata)*

Fifteen models of each of the proteins (PrA, PrB, and Atg8) from *S. cerevisiae* and the fungal phytopathogens were generated by homology modeling, selecting in each case the best model based on its DOPE potential. The overlap of the PrAs of different organisms is shown in **Supplementary Figure 1**, while the overlap of the Atg8s and PrBs is included in the main text.

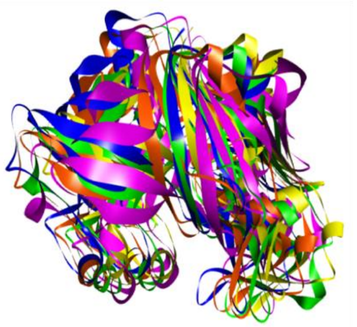

Supplementary Figure 1. Overlap of the best models of PrA of *S. cerevisiae* and the three fungal phytopathogens, represented as flat ribbons. PrAHs (orange), PrASc (green), PrBUm (blue), PrBMo (fuchsia), and PrBAa (yellow).

Protein-peptide interactions

Considering the similarity between the endogenous inhibitor of *S. cerevisiae* PrB (PBI2) and that of the *U. maydis* homolog (Um10059), as well as between the two respective propeptides (PrB1 and Um4400) **(Supplementary Figure 2)**, a protein-protein interaction study was carried out between the PrBs analyzed presently and the PBI2 inhibitor of *S. cerevisiae*, the Um10059 of *U. maydis*, and the PrB1 and Um4400 propeptides.

For the purpose of clarity, only the interactions between Um10059 and the PrBs are shown in the main text. The results of PBI2 and the propeptides are illustrated in **Supplementary Figures 3, and 4and Supplementary Table 2.**

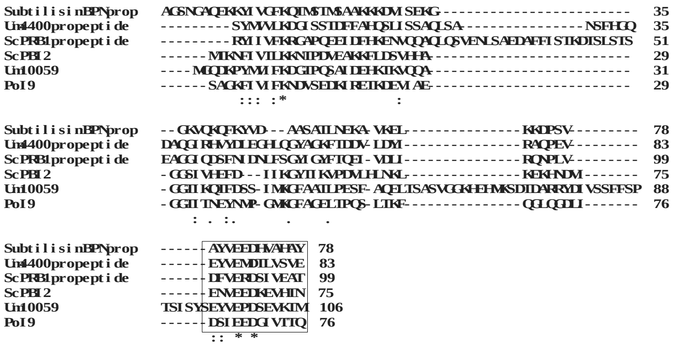

Supplementary Figure 2. Sequence alignment of the inhibitors and propeptides of the serine endopeptidases. The sequences of ScPrB, UmPrB, subtilisin BPN´(1SPB), PBI2 (YNL015W), POIA1 (*Pleorotus ostreatus*, Q7M4T6), and peptide Um10059 were aligned in Clustal W. The box highlights the C-terminal domain.

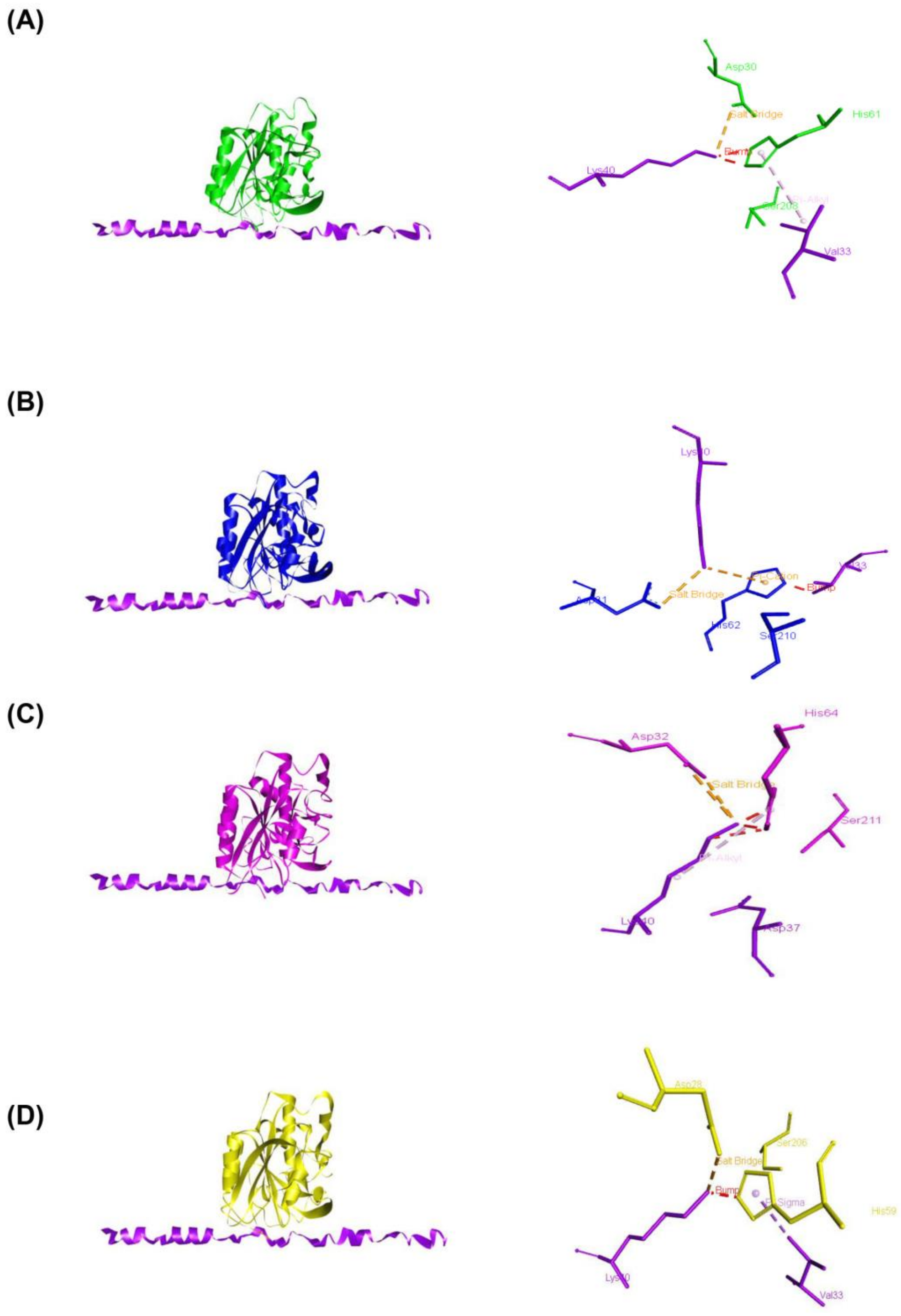

Supplementary Figure 3. Intermolecular interactions of the inhibitor PBI2 (purple) with PrBSc (**A**), PrBUm (**B**), PrBMo (**C**), and PrBAa (**D**). Dotted lines indicate the type of interactions. For the purpose of clarity, only the interactions between the amino acid residues of the PrB catalytic triad (Asp, His, and Ser) and PBI2 are depicted.

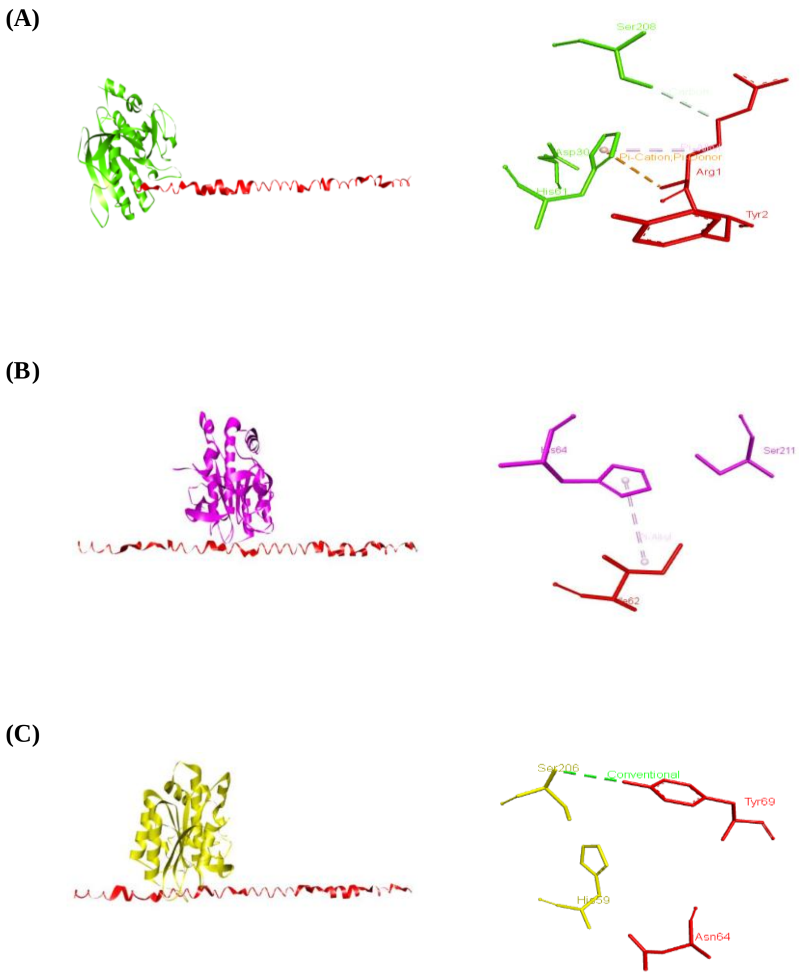

Supplementary Figure 4. Intermolecular interactions of the PrB propeptide (red) with PrBSc (**A**), PrBMo (**B**), and PrBAa (**C**). Dotted lines indicate the type of interactions. For the purpose of clarity, only the interactions between the amino acid residues of the PrB catalytic triad (Asp, His, and Ser) and the PrB inhibitor are shown. Note: The interactions with *U. maydis* are not included because none of the residues of the catalytic triad interact with the inhibitor of this fungus.

Supplementary Table 2

Receptor-ligand interface residues (Um4400-propeptide)

**PrBSc residue**	**propeptide residue**	**PrBUm residue**	**propeptide residue**
**Thr69**	His70	Thr71	His70
**Lys74**	Phe69	Lys76	Phe69
**Tyr75**	Ser65, Phe69, His70	Tyr77	Ala66, Phe69, His70,
**Gly76**	Ala66		Asp73
**Val77**	Al62,Gln63, Ala66	Gly78	Ala66
**Lys79**	Ser65	Val79	Ala62, Ala66
**Asn193**	His70	Lys81	Ser65, Ala66
**Ile194**	His70	Asn195	His70
**Ser195**	His70, Asp73, Ala74	Ile196	His70
**Thr196**	His70	Ser197	His70, Asp73, Ala74
**Val203**	Asp73, Ile77	Thr198	His70
**Asn204**	Ile77	Lys203	Ile77
**Thr205**	Ala74	Thr204	Asp73
**His215**	Thr63	His216	Gln63
**Gly218**	Ile59	Gly219	Ile59
**Leu219**	Ile59, Thr63	Leu220	Gln63
**Tyr222**	Ilr59	Leu221	Ile59
**Leu223**	His55, Ile59	Tyr223	Ile59
**Ile226**	His55	Tyr224	His55, Gln56, Ile59
**Tyr227**	Phe52	Leu227	His55
**Leu230**	Phe52	Gln228	Phe52, His55
**Asp238**	Leu58	Ile231	Phe52
**Ser242**	Gln56	Lys235	Phe52
**Lys245**	Gln56	Asp239	Phe52
**Leu246**	Gln56, Ser60, Gln63	Lys242	Phe53, Gln56
		Val243	Gln56, Gln63

Supplementary Table 2 (continued)			
**Receptor-ligand interface residues (Um4400-propeptide)**			
**PrBAa residue**	**propeptide residue**	**PrBMo residue**	**propeptide residue**
**Thr66**	Phe53	Thr71	Gln63
**Tyr72**	Phe53,His55,Gln56	Lys76	Leu64
**Gly73**	Phe53	Tyr77	Gln63
**Val74**	Ser49, Phe53	Gly78	Gln63
**Leu168**	Ala54	Val79	Gln63, Asn67
**Pro187**	Thr50	Lys81	Leu64
**Leu189**	Ser57	Ser171	Ile59
**Asn190**	Gln56, Ser57, Ser60,	Leu173	Ala62
	Leu64	Pro192	Ala62
**Ile191**	Phe53, Gln56, Ser57	Gly193	Ile59
**Ser192**	Gln56, Ile59, Ser60	Leu194	Ile59
**His199**	Gln63	Asn195	Phe52, His55, Gln56, Ile59
**Ala200**	Gln63		Ile59, Gln63
**Thr201**	Ile59, Ser60,	Ile196	Gln53, Gln56
	Gln63,Leu64	Ser197	Gln56
**Asn202**	Ser60	Thr204	Gln53
**Met207**	Phe53	Ala205	Phe52
**His211**	Ser49, Thr50, Phe52,	Ile206	Gln63, Ala66
	Phe53	His216	His70
**Leu215**	Val42, Asp45, Gly46	Ile217	Ala66, His70
**Tyr218**	Asp45	Leu220	His70, Asp73, Ala74
**Met219**	Tyr39, Val42	Tyr224	Ala74
**Leu222**	Ser38, Val41, Val42	Leu227	Arg78
**Gln223**	Ile35, Ser38, Tyr39	Gln228	Ile77
**Ile226**	Tyr39	Lys 235	Ile77
**Lys230**	Tyr39	Asp239	His70
**Asn234**	Tyr39	Leu240	Asp73
**Ser237**	Leu43	Lys242	His70
**Val238**	Gly46, Ile47	Val243	

References

Aiyar, A. (2000). The use of CLUSTAL W and CLUSTAL X for multiple sequence alignment. *Methods Mol. Biol. (Clifton, N.J.),* 132, 221–241. https://doi.org/10.1385/1-59259-192-2:221

Berman, H. M., Westbrook, J., Feng, Z., Gilliland, G., Bhat, T. N., Weissig, H., Shindyalov, I. N., and Bourne, P. E. (2000). The Protein Data Bank. *Nucleic. Acids. Res.*, 28(1), 235–242. https://doi.org/10.1093/nar/28.1.235

Morrison, D. A. (2007). Increasing the efficiency of searches for the maximum likelihood tree in a phylogenetic analysis of up to 150 nucleotide sequences. *Sys. Biol.* 56(6), 988–1010. https://doi.org/10.1080/10635150701779808

O'Boyle, N. M., Banck, M., James, C. A., Morley, C., Vandermeersch, T., and Hutchison, GR. (2011). Open Babel: An open chemical toolbox. *J. Cheminf.* 3, 33. https://doi.org/10.1186/1758-2946-3-33

Posada, D. (2008). jModelTest: phylogenetic model averaging. *Mol. Biol. Evol.*, 25(7), 1253–1256. https://doi.org/10.1093/molbev/msn083

Sharma, S., Ciufo, S., Starchenko, E., Darji, D., Chlumsky, L., Karsch-Mizrachi, I., and Schoch, C. L. (2018). The NCBI BioCollections Database. *J. Biol. Databases and curation*. https://doi.org/10.1093/database/bay006

Shen, M. Y., and Sali, A. (2006). Statistical potential for assessment and prediction of protein structures. *PRCIEI*, 15(11), 2507–2524. https://doi.org/10.1110/ps.062416606

Tamura, K., Stecher, G., and Kumar, S. (2021). MEGA11: Molecular Evolutionary Genetics Analysis Version 11. *Mol. Biol. Evol.*, 38(7), 3022–3027. https://doi.org/10.1093/molbev/msab120

Tamura, K., Stecher, G., Peterson, D., Filipski, A., and Kumar, S. (2013). MEGA6: Molecular Evolutionary Genetics Analysis version 6.0. *Mol. Biol. Evol.*, 30(12), 2725–2729. https://doi.org/10.1093/molbev/mst197

Webb, B., and Sali, A. (2014). Comparative Protein Structure Modeling Using MODELLER. *Curr. Prot. Bioinform.*, *47*, 5.6.1–5.6.32. https://doi.org/10.1002/0471250953.bi0506s47

Yan, Y., Tao, H., He, J., and Huang, S. Y. (2020). The HDOCK server for integrated protein-protein docking. *Nat. Protoc*., 15, 1829–1852 https://doi.org/10.1038/s41596-020-0312-x
